# Synthesis and Effect of Structure on Swelling Properties of Hydrogels Based on High Methylated Pectin and Acrylic Polymers

**DOI:** 10.3390/polym11010114

**Published:** 2019-01-10

**Authors:** Grzegorz Kowalski, Karolina Kijowska, Mariusz Witczak, Łukasz Kuterasiński, Marcin Łukasiewicz

**Affiliations:** 1 University of Agriculture in Krakow, Faculty of Food Technology, ul. Balicka 122, 30-149 Kraków, Poland; karolina1.kijowska@gmail.com (K.K.); mariusz.witczak@urk.edu.pl (M.W.); rrlukasi@kinga.cyf-kr.edu.pl (M.Ł.); 2 Jerzy Haber Institute of Catalysis and Surface Chemistry Polish Academy of Sciences, ul. Niezapominajek 8, 30-239 Krakow, Poland; nckutera@cyf-kr.edu.pl

**Keywords:** hydrogels, poly(acrylic acid), pectin, swelling, DSC

## Abstract

The aim of the research was to develop new pectin-based hydrogels with excellent swelling properties. Superabsorbent hydrogels composed of high methylated pectin and partially neutralized poly(acrylic acid) was obtained by free radical polymerization in aqueous solution in the presence of crosslinking agent—*N*,*N*’-methylenebisacrylamide. The effect of crosslinker content and pectin to acrylic acid ratio on the swelling properties of hydrogels was investigated. In addition, the thermodynamic characteristic of hydrogels was obtained by DSC. Furthermore, the structure of pectin-based hydrogels was characterized by FTIR and GPC. It was also proved that poly(acrylic acid) is grafted on pectin particles. The results showed that introduction of small amount of pectin (up to 6.7 wt %) to poly(acrylic acid) hydrogel increase the swelling capacity, while further increasing of pectin ratio cause decrease of swelling.

## 1. Introduction

Hydrogels have been used in many fields, they are widely used in agriculture [1,2,3], pharmaceutical industry [4,5], food [6], in personal hygiene [7], and in biomedicine [6,7,8,9]. These type of hydrogels are often used as biosensors [10] as well as in disposable diapers, sanitary napkins, plant irrigation regulators [3], wastewater treatment [11], and for blocking cracks and leaks. In the food industry, they can act as food additives [12], or inserts that absorb leaking liquids. In medicine, hydrogel-based matrices are used for the controlled release of drugs and enzymes [4], for the construction of artificial organs as well as contact lenses.

There is currently a growing interest on the development of new hydrogel materials. Hydrogels on the base of synthetic polymers associated with biopolymers (mainly carbohydrates) have attracted extensive attention of the scientists in the field of polymer science for pharmaceutical, biomedical, and food applications due to the unique properties e.g., high swelling, potential biocompatibility, and ecofriendly nature [13,14,15,16,17,18]. Superabsorbent hydrogels, relative to their own mass can absorb and retain extraordinary large amounts of water or aqueous solution [19]. These ultrahigh absorbing materials can absorb deionized water as high as 10–1000 g/g whereas the absorption capacity of common hydrogels is not more 1 g/g.

Carbohydrates are characterized by high hydrophilicity and biocompatibility. Furthermore, these kinds of materials are sometimes called ‘green composites’ or ‘bio based polymers’ primarily due to the biodegradability and renewability of their resource. The combination of advantages of biopolymers with high processability and stability of the synthetic polymers allows to obtain functional biomaterials [20]. What is more their properties depend on the selection of the substrate, the quantitative ratio and applied modifications. For this reason it is possible to develop the material based on the same substrates but showing different properties [21].

Pectin is a complex heteropolysaccharide with high molecular weight and polyanionic nature. Hydrophilic character and the ability to create gels are main properties of pectin that find widespread use in the food industry. Those functional properties of pectin depend mainly on the source, molecular weight and degree of esterification (DE). Having regard to its functional properties like non-toxicity, biocompatibility, possibility to carrying signal molecules and supporting various biologically active substances pectin has potential in biomedical applications [22].

The polyanionic character causes its high capacity absorption and pH-responsive properties. Pectin has potential in the drug delivery systems [23]. Combining the unique properties of pectin with features of the synthetic superabsorbents, e.g., poly(acrylic acid) could be developed to form the hybrid hydrogel material, which could be defined as three-dimensional hydrogel network composed of synthetic polymer and biopolymers [24]. There are some reports on the use of pectin based hydrogels grafted with vinyl monomers as acrylamide [25,26], acrylamide and acrylic acid [27], N-vinylpyrrolidone [28]. Pectin/Poly(sodium acrylate) hydrogels synthesized in molds were also investigated [15], in contrast to our studies, which were carried out on hydrogels in powder form. There are some researches about new hydrogel materials however very important seems to be optimization of the synthesis process to obtain product with desired properties.

In our study, we report the synthesis and characteristic of poly(acrylic acid) hydrogels (AA-SA) based on high methylated citrus-apple pectin with *D*_e_ = 0.67 obtained via a free radical polymerization. The parameters of formed gel network significantly depend on polymerization conditions such as monomer and pectin concentration as well as cross-linker content. In this study, we focus on investigating how above factors influence on swelling parameters. Phase transitions of the hydrogels were also analyzed. In addition structure of pectin/poly(acrylic acid) hydrogels was also examined to clarify way of reaction.

## 2. Materials and Methods

### 2.1. Materials

In this work pectin methylation degree (*D*_e_ = 0.67) (Pektowin Jasło, Poland) was used. Acrylic acid (AA) and *N*,*N*’-methylenebisacrylamide (MBA) were purchased from Fluka (Buchs, Switzerland). Potassium persulfate citric acid, sodium hydrogen phosphate, methanol, isopropanol, and NaOH were purchased from POCH (Gliwice, Poland). All reagents were of analytical grade.

### 2.2. Pectin-Based Hydrogel Synthesis

Aqueous solutions of the acrylic acid monomer 30 g (0.416 mol) dissolved in 10 g of water were partially neutralized with 5 g (0.125 mol) of sodium hydroxide dissolved in 14.5 g of water. This means that 30% of the carboxyl groups has been neutralized and is in the form of sodium salt. Then *N*,*N*’-methylenebisacrylamide solution 0.0098 g (0.0633 mmol) in 3.2 g of water and potassium persulfate (0.12 g in 3.2 g of water) were prepared. The pectin solution dissolved in 100 g of distilled water was placed in a 500 mL three-necked flask and heated in a water bath to 80 °C. Hydrocolloid solution was mixed continuously using a mechanical stirrer with rotation controlling at 500 rpm. Reaction was carried out at nitrogen atmosphere to minimize the risk of potential side reactions. Then solution of acrylic acid, sodium acrylate and an appropriate amount of *N*,*N*’-methylenebisacrylamide (cross-linking agent) was added. After obtaining a constant temperature (80 °C), a solution of potassium persulfate was added. Time of the initiator addition determines the beginning of the free radical reaction. After a few minutes of reaction mixture viscosity rapidly increases and gel is formed. After gelation, the samples of the hydrogels were still kept in a water bath at a temperature of 80 °C, under nitrogen, so the polymerization reaction took place completely. The total reaction time was 60 min. After the reaction was completed, the mixture was allowed to cool at room temperature and were washed several times with large amount of 80:20 methanol:water mixture. After removing of methanol, the obtained gel was cut into small pieces and dried in an oven at 60 °C for 48 h. Hydrogel samples were transferred to Petri dishes and heated in an air-circulating oven at 60 °C for minimum 48 h to dryness. The dried gels were ground by mini grinder (laboratory scale) and screened. The classified particles were stored in tightly-stoppered plastic containers and used in further experiments.

### 2.3. Sol Content Determination

To a 1 l beaker containing 100 mL of 1.0% sodium chloride solution, 0.1 g (± 0.0001 g) of dried superabsorbent was poured. The hydrogel suspension was allowed to shake for 72 h, so that the soluble fraction went to the solution. After that suspension was filtered and UV/VIS analysis was performed at the range 180–300 nm in a single-beam spectrophotometer (Labomed, Inc., Los Angeles, CA, USA), UV-VIS 2800). The measurements were carried out in a quartz cuvette with a path length of 1 cm. As a reference 1% aqueous NaCl solution was used. Calibration of the apparatus was made with acrylic acid solutions at fixed concentrations. Maximum of absorption for the tested samples was observed at 208 nm.

### 2.4. Molecular Weight and Molecular Mass Distribution of Water Soluble Fraction

For a detailed analysis of the soluble fraction of the hydrogel samples, it was isolated by continuous extraction in a Soxhlet apparatus with water for 24 h. After the process was completed solvent was evaporated and extracts were dried in an oven at 45 °C. For chromatographic analysis, extract solutions with concentration ca. 1 mg/mL were prepared. Molecular weight distributions were performed by means of gel permeation chromatography (GPC). The system of three columns Ultrahydrogel 2000 (Waters, Milford, MA, USA), Ultra Hydrogel 500 (Waters) and Ultrahydrogel 120 (Knauer, Germany) connected in series with the RI detector (Knauer, Berlin, Germany) was used. As an eluent 0.1 mol/L NaNO_3_ solution in water was applied. The measurement was carried out at 25 °C, at a flow rate of 0.6 mL/min and sample volume of 100 μL was injected. A calibration using pullulan standards (Shodex, Yokohama, Japan) was performed.

### 2.5. FT-IR Spectroscopy

The FT-IR experiments were carried out using Nicolet 6700 spectrometer with DTGS detector from Thermo Scientific. The scanning range of the IR spectra was 400–4000 cm^−1^ and 64 scans were obtained for each spectrum. Studied samples were in KBr wafer form with a diameter of 1.3 cm, and mass 300 mg. The wafers were obtained under 5 Mg for 1 min, and contained ca 1 mg of studied sample in powder form. The FT-IR spectra are given in Figure 7.

### 2.6. Swelling Properties

A sample of about 0.2 g of dried hydrogel was immersed in excess of distilled water (200 mL) at room temperature for defined period of time. During swelling process samples were slightly stirred with magnetic stirrer (300 rpm). The dispersion of water-swollen gel particles was carefully filtered through Büchner funnel to decrease filtration time. Filtered samples were weighed. For measuring absorption rate, the samples were removed from water after precisely defined period of time. For each sample, measurements were repeated three times. The swelling ratio was calculated using Equation (1)

(1) $$W_{t}=\frac{M_{w}-M_{d}}{M_{d}}$$

where

*W*_t_—swelling ratio at time *t* [g H_2_O/g dry gel]
*M*_w_—weight of the swollen hydrogel at time *t* [g]
*M*_p_—weight of the dried gel [g]

Changes in swelling properties were estimated with the following kinetic Equation (2)

(2) $$W\left(t,\;x\right)=W_{\infty }\frac{k_{1}\cdot t}{{\left(1+K\cdot t\right)}^{a}}=W_{\max}\cdot e^{-b\cdot x}\frac{k_{1}\cdot t}{{\left(1+K\cdot t\right)}^{a}}$$

In this equation, *x* represents molar ratio of crosslinker to monomer, *k*_1_ represents the water swelling rate constant by hydrogel (g/(g∙s)), while $W_{\infty }$ represents water absorption at the equilibrium. Parameters *K* (s^−1^) and α exponent determine the denominator value, which represents the resistance of the diffusion process. Estimation of the parameters of Equation (2) was carried out by Marquardt–Levenberg method.

### 2.7. Thermal Characterization

Thermodynamic characteristics of analyzed hydrogels was obtained using differential scanning calorimeter DSC 204F1 Phoenix (Netzsch, Selb, Germany). Hydrogel samples (~15 mg) was preheated in DSC aluminum pan to 140 °C, then cooled to approx. 50 °C (10 deg/min) below predicted *T*_g_ value and finally heated to 250 °C (10 deg/min). DSC measurements were made at nitrogen atmosphere.

On the basis of registered thermograms the onset (*T*_onsetg_), medium (*T*_midg_), inflection (*T*_infg_), endset (*T*_Eg_) glass transition temperatures and onset (*T*_onsetm_), peak (*T*_pm_), endset (*T*_endm_) melting point temperatures as well as change in thermal capacity Δ*c*_p_ and melting heat ΔH were determined using the Proteus Analysis software (Netzsch, Germany). As the glass transition temperature, the medium temperature (*T*_midg_) of the transformation, hereinafter referred to as *T*_g_, was assumed. The DSC analyzes were performed in duplicate.

### 2.8. Statistical Analyses

In order to establish the statistical differences between means the date were treated by one-factor analysis of variance, and the least significant difference (LSD) using Fisher test at significance level 0.05 was calculated. The influence of selected factors was analyzed with the use of two-factor analysis of variance. In order to show the relationship between the composition of the mixture and the parameters characterizing the product, Pearson correlation coefficients were calculated In order to show the relationship between mixture composition and parameters characterizing the product, Pearson correlation coefficients were calculated. All calculations were performed with statistical software package Statistica 12.0 (StatSoft Inc., Tulsa, OK, USA).

## 3. Results and Discussion

As part of the research described in this article, the synthesis of hybrid hydrogels characterized by very high swelling capacity was carried out. Hydrogels based on natural and synthetic polymers were obtained. High methylated pectin (*D*_e_ = 67%) and poly(acrylic acid) partially present in the form of the sodium salt were used. The hydrogel was obtained by free radical polymerization reaction of acrylic acid, sodium acrylate with simultaneous grafting of the resulting polymer on pectin. The polymerization/grafting reaction was carried out in the presence of a small amount of cross-linking agent N,N’-methylenebisacrylamide (MBA) to obtain a cross-linked structure of poly(acrylic acid) and pectin. In our research, the hydrogel synthesis reaction was carried in a controlled way. Two series of hydrogels with different amounts of cross-linking agent were obtained ranging from 0.0048 to 0.0804 g (Table 1, Samples P1–P6) and variable pectin content 0–10 g (Table 1, samples P3A-P3G) in the reaction mixture. An attempt was made to evaluate the influence of polymerization reaction parameters on the properties of the final product, i.e., swelling capacity and kinetic of swelling of hydrogels. Determination of amount and molecular weight of hydrogels soluble fraction was investigated as well as glass transition and melting characteristic temperatures.

### 3.1. Swelling Characteristic of Poly(Acrylic Acid)/Pectin Hydrogels

Swelling properties of hydrogels were determined by placing of dried hydrogels in distilled water for a specific time of 1, 2, 3, 5, 10, 20, 60, and 1440 min, then water which was not absorbed was removed from the samples by the filtration.

In our research, the effect of two important parameters on the water absorption of superabsorbents, i.e., the amount of cross-linking agent in the reaction mixture, as well as the amount of pectin used in the reaction were determined.

**Figure 1:**
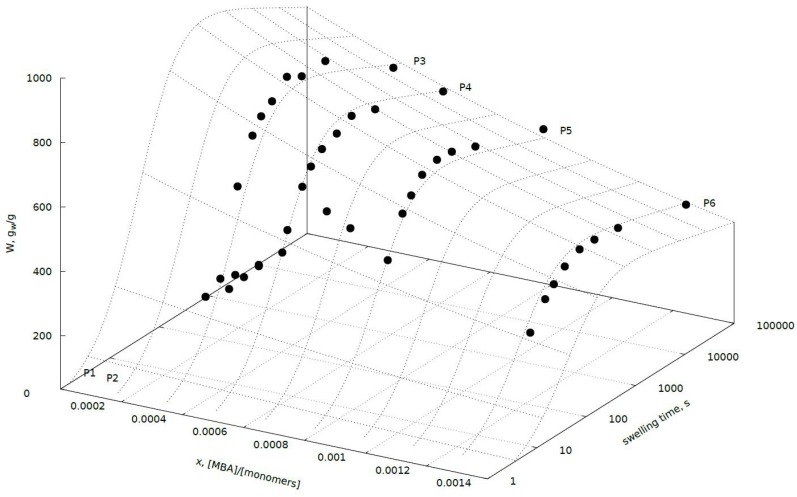
Swelling capacity as the function of crosslinker to monomer ratio *x* and swelling time *t* of pectin-based hydrogels.

The effect of the crosslinker concentration in the reaction feed was studied by varying the molar ratio of MBA to AA (*x*) from 0.000075 to 0.001256 and keeping all other synthesis parameters constant (Table 1). Figure 1 shows the results of the swelling kinetic study of this group of hydrogels. Changes in swelling properties were estimated with the kinetic Equation (2). P1 and P2 samples were placed on Figure 1, but they were not taken into account for the estimation of the swelling kinetics parameters of the Equation (2) by Marquardt–Levenberg method. The behavior of these two samples during the experiment completely differed from the entire group of remaining hydrogels. These two reactions were characterized by a very low amount of cross-linking agent, which turned out to be insufficient to obtain stable hydrogels, able to absorb and maintain the water molecules in their structure. To be sure hydrogels P1 and P2b were able to absorb the water, but the water was very weakly bonded and during removing of the water excess by filtration it was very easy to remove the water absorbed by these hydrogels. Summing up the amount of crosslinker used in the synthesis of P1 and P2 gels MBA to AA ratio (*x*) 0.000075 and 0.000152 respectively appears to be too low to obtain stable hydrogels.

As the crosslinker concentration in the initial feed increases swelling degree decreases and could be fitted by the following Equation (3)

(3) $$W\left(t,\;x\right)=300\cdot e^{-534.2\cdot x}\frac{0.050\cdot t}{{\left(1+0.022\cdot t\right)}^{1.053}}$$

In the tested hydrogels, water absorption in equilibrium is variable in the range of 380–610 g/g. Quantity of absorbed water depends on the amount of cross-linking agent used in the reaction, and as expected, decreases with increasing amount thereof. Hydrogels prepared in the presence of a larger amount of cross-linking agent are characterized by a lower rate of water absorption.

This phenomenon can be easily explained by the diffusion of water into the hydrogel network with high cross-linking density which is difficult. This is related to the fact that the hydrogel structure with high cross-link density is more compact and the mesh size in the network is much smaller. The calculated time constants of water absorption process are shown in Equation (3). First of all, the hydrogels varied in values *W*_∞_, which it the highest for P3. The value of *W*_∞_ was dependent on *x* (Equation (2)), the molar ratio of crosslinker to monomer. For samples P3-P6, the higher the *x* value the lower the water absorption observed. The water absorption rate constant is 0.050 s^−1^, and is independent of the molar ratio of crosslinker to monomer *x*. Inhibition constant *K* is 0.022 s^−1^ and is independent of the molar ratio *x* as well. The value of the exponent corresponds to resistance of water absorption process and is a little bit larger than unity. It is in the denominator of the water absorption rate equation, which points to high diffusion resistance during swelling process. Therefore, the ability of swelling in water is determined mainly by the crosslinking density in hydrogel structure.

Tendency of swelling degree decreasing at equilibrium with increasing the crosslinker concentration is in agreement with the Flory theory and with the result of other authors [29,30,31,32].

Research was also carried out to determine the effect of the pectin/monomer ratio on the properties of the resulting hydrogel. Analyzing the water absorption kinetics curves, specific maxima of water absorption after 20 min can be observed. Then there is a slight decrease in swelling capacity for all samples, until equilibrium after 24 h was reached. The slight decrease in water absorption of hydrogels after 1 h can be explained in two ways: a) lowering the pH of the environment during the process [33], b) changes in pectin configuration. 

During the absorption of water, the polymer network expands. Due to expanding hydrogel network and dissociation of acid molecules, the pH of the solution decreases and as result decreasing in water absorption is observed globally. It could not be ignored that hydrogels may still contain minor amounts of oligomers which were not bound with the polymer network and could be released into the surrounding environment.

It cannot be also excluded that changes in the molecules of the pectin occurs. However, this case seems to be rather negligible, since the reduction of water absorption is also observed in the hydrogel which not contain pectin (P3A). Additionally, further increase the pectin content in the hydrogel causes that the observed decrease in water absorption between 1 and 24 h is much smaller, it is particularly visible for the P3G sample.

**Figure 2:**
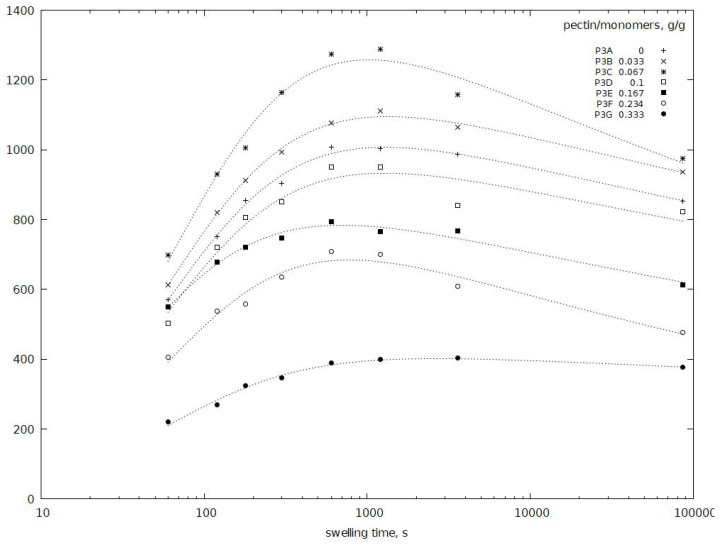
Swelling capacity as the function of swelling time *t* for hydrogels with various ratio of pectin to monomers (P3A–P3G).

Analyzing the effect of pectin content on water absorption of the obtained hydrogels (Figure 2), it can be seen that a small addition of pectin in the range of 3.3–6.7% (P3B and P3C) increases swelling capacity compared to partially neutralized poly(acrylic acid). Swelling capacity for these hydrogels at equilibrium is 950–980 g/g, depending on the content of pectin. The rate of absorption of water in the initial absorption period is also much higher. Further increase of pectin content in the hydrogel above 6.7% causes a gradual decrease in swelling capacity of hydrogels. It reaches the smallest value (370 g/g) for 33.3% pectin content for P3G.

The presence of pectin in the hydrogel structure causes an increase in water absorption but further increase of pectin content above 6.7% causes a deterioration of these properties. This phenomenon may be associated with a significant increase in the viscosity of the reaction mixture as concentration of pectin increases. As a result, contact of reagents is difficult and the resulting structure may not have the preferred form of polymer network.

### 3.2. Analysis of Sol Content in Pectin-Based Hydrogels

**Figure 3:**
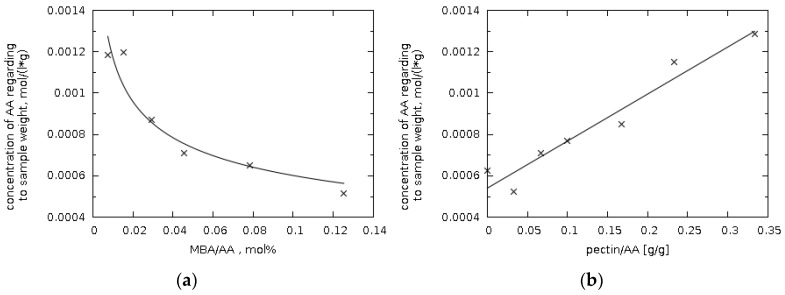
Concentration of unreacted acrylic acid depending on the amount of (**a**) cross-linking agent (MBA); (**b**) pectin in the hydrogel samples, with reference to 1 g of hydrogel.

Due to the fact that the cross-linking reaction does not occur completely, the soluble hydrogel fractions were analyzed using UV–vis spectroscopy (analysis of the band which is typical for carbon–carbon double bonds) [34]. The presence of soluble fraction is caused by the fact that some of the formed polymeric chains do not incorporate into the polymer network. Therefore, they can be easily extracted and constitutes a soluble fraction. It is worth pointing out that only insignificant amount of unreacted acrylic acid or soluble short-chain acrylic acid oligomers have been confirmed, in which C=C double bonds could be present. Since the pectin molecules are less mobile than the acrylic acid molecules, the possibility of the reaction with acrylic acid depends on the distance of the acid molecules to the hydroxyl groups of pectin [16]. Based on the experiment, it can be concluded that increasing the amount of the crosslinking agent has a positive effect on the conversion of acrylic acid. A decrease in the number of soluble fractions containing C=C double bonds was observed, along with an increase in the amount of MBA (Figure 3a) [35]. It was also shown that this relationship is of a power nature [36].

In the case of a series of samples with a variable amount of pectin (P3A-P3G), an increase in the amount of unreacted AA is observed with an increase in the pectin content in the reaction mixture (Figure 3b). Such behavior may indicate that, despite the increase of hydroxyl groups amount where possibly reaction with AA could occur propagation reaction of polymeric chains is preferred. Another important factor is fact that a higher concentration of pectin causes an increase in the viscosity of the reaction mixture, resulting in a difficult mixing. As a result, there is a decreasing of probability of substrates interaction, and consequently a reduction in the conversion rate.

The content of soluble fractions can affect the initial water absorption rate, although the rate also depends greatly on the maximum capacity of the water absorbed by a specific hydrogel. This behavior can be attributed initially to a high chemical potential or osmotic driving force for swelling due to the presence of a substance dissolved in the gel [37].

### 3.3. GPC Analysis of the Soluble Fraction

Dried aqueous extracts of hydrogel samples were subjected to GPC chromatographic analysis. Deep analysis of chromatograms may indirectly give information on the composition of cross-linked and insoluble fractions responsible hydrogel.

**Figure 4:**
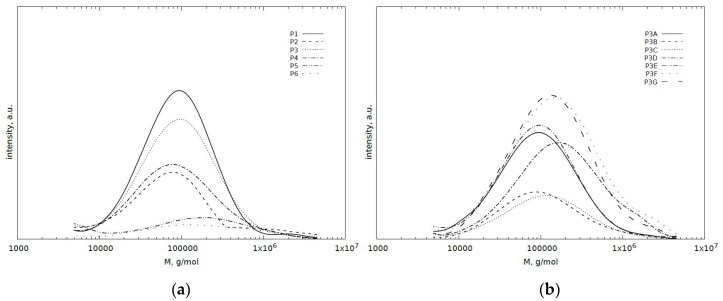
Molecular mass distribution for soluble fractions of pectin based hydrogels depending on (**a**) amount of cross-linking agent; (**b**) amount of high methylated pectin.

The molecular weight distribution profiles are characterized by relatively high values of the average molecular weights, the number average molar mass is in the range of 0.44×10^5^–0.84×10^5^ g/mol, and the mass average is in the range 0.92×10^5^–3.45×10^5^ g/mol. Water-soluble fractions also have a relatively high polydyspersity index (from 2.3 to 4.6) (Table 2). These features indicate the presence of polymeric compounds. However, due to the significant difference between the average molecular weights of these extracts and the pure pectin (*M*_n_ = 0.88 × 10^5^ and *M*_w_ = 7.6 × 10^5^ g/mol, Ɖ = 8.6), it can be concluded that the extracted polymers are not pectin. Furthermore, relatively low molecular weight and polydyspersity of polymers presented on GPC spectra peaks (Figure 4), which are related to the presence of non-crosslinked and soluble fragments of poly(acrylic) acid chains suggests that these polymers do not contain pectin. Accordingly, it can be assumed that pectin has been incorporated into crosslinked poly(acrylic acid) chains. For hydrogels with a variable content of cross-linking agent (P1–P6), only slight effect on the composition of the soluble fraction was observed. Increasing the pectin content initially increases the number and weight average molecular weight of soluble fractions, with the maximum values for the sample P3D, further increase of the pectin content in the hydrogel causes a decrease in molecular weight.

### 3.4. Thermal Analysis

**Figure 5:**
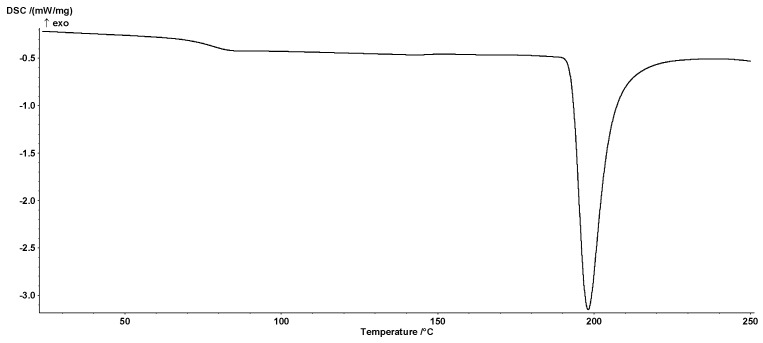
Typical DSC curve of analyzed sample (second scan)—sample P3B.

Figure 5 shows an example curve for pectin-based hydrogels obtained using DSC. In the studied range, the curves were characterized by the occurrence of glass transition (Table 3) and melting (Table 4) characteristic peaks. 

The highest value of temperatures associated with the glass transition were characterized by the crosslinked sample without the presence of pectin. The increase of pectin amount caused a drop in all temperatures characterizing the glass transition. The exception was the value of the transformation range, the value of which increased with the increase of pectin amount in the reaction mixture. Although the relationship was not linear, the obtained values of correlation coefficients between the values of characteristic temperatures (*T*_ong_, *T*_midg_, *T*_infg_, *T*_endg_) were statistically significant and ranged from −0.604 to −0.768 with *p* values below 0.007.

**Figure 6:**
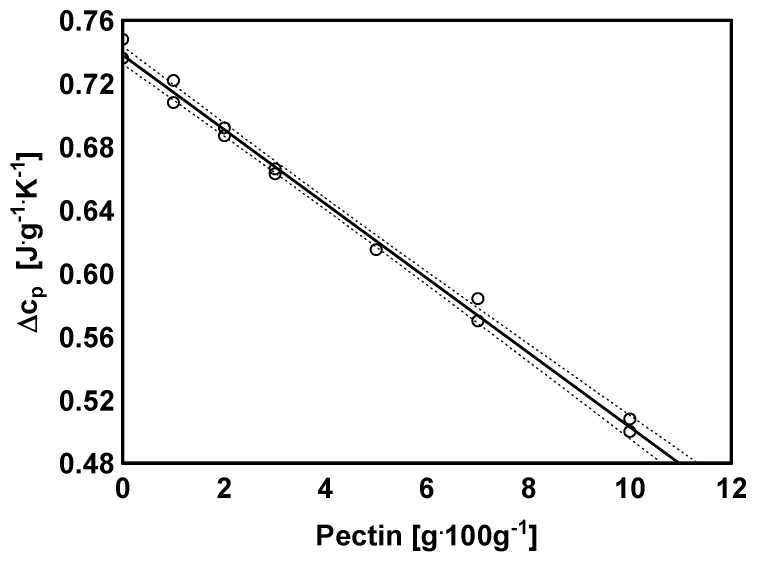
Thermal capacity changes depending on the pectin content (○—experimental data, –––—calculated model, ----—95% confidence interval)).

Relatively good representation of the data was obtained using the exponential model (data not presented) for which *R*^2^ was 0.947. In the case of the range and change of the thermal capacity, strong correlations between the pectin contribution and the analyzed parameters were found (Figure 6). The correlation coefficient (R) between the transformation range and the pectin amount was 0.962 (*p* < 0.001). However, R value between the Δ*c*_p_ (Figure 6) and the pectin amount was −0.997 (*p* < 0.001). This indicates the determining effect of pectin on the changes in the characteristic values of glass transition.

Similarly, statistically significant correlations were found between the transformation range and changes in thermal capacity as well as amount of unreacted acrylic acid (0.974 and −0.999, respectively, at *p* < 0.001). The simultaneous strong relationship between the range of glass transition and the pectin amount as well as the changes in thermal capacity and the amount of unreacted acid was observed. This phenomenon is due to the strong relationship between the amount of pectin and unreacted acrylic acid (*r* = 0.970, *p* < 0.001). There was no relationship between the amount of crosslinking agent and the values of the parameters characterizing the glass transition process. The analysis of the dependencies indicates a small variation only—the initial increase, then the decrease and finally increase with the highest amount of the crosslinking agent. The resulting variation (statistically significant) indicates that the *T*_g_ value is related to the crosslinking degree. This dependence is determined by the cross-linking reaction, which results of high variability in the parameters characterizing the obtained hydrogel and affecting the glass transition temperatures as well.

There was a strong correlation between the pectin amount, the enthalpy value (0.938, *p* < 0.001) and the melting process temperatures of *T*_onsetm_, *T*_pm_, *T*_endm_ (−0.815, −0.836, −0.815 at *p* < 0.001, respectively). The lowest value of Δ*H* and at the same time the highest transformation temperatures was characterized sample obtained without the addition of pectin. Enthalpy values increased, whereas characteristic temperatures decreased with increasing of polysaccharide in the reaction mixture. Similarly, as in the case of glass transition, the melting peak showed no significant correlation between the amount of cross-linking agent and the values of the characteristic temperatures of melting process.

### 3.5. FT–IR Spectra of Poly(Acrylic Acid)/Pectin Hydrogels

**Figure 7:**
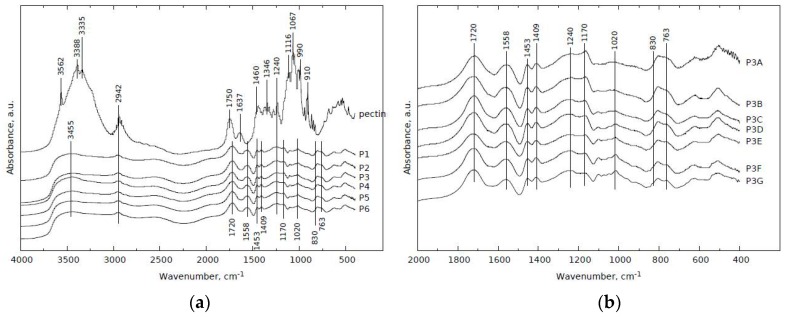
FT–IR spectra of pectin-based hydrogels depending on (**a**) amount of cross-linking agent; (**b**) amount of high methylated pectin.

Figure 7a shows the IR spectra of the mixture of pectin, crosslinker and AA-SA copolymer with different chemical composition (line a–g). The content of pectin was constant, meanwhile the amount of crosslinker have been changed gradually. 

In the case of pure pectin (line pectin), the bands at 3562, 3388, and at 3335 cm^−1^ can be assigned to OH stretching mode, while the signal observed at 2942 cm^−1^ might be attributed to stretching vibrations of C–H groups. The distinct band registered at 1750 cm^−1^ corresponds with an asymmetric C=O stretching vibrations present in carboxyl or COOCH_3_ groupings. The bands at ca 1637 and 1460 cm^−1^ may be assigned to COO^-^ symmetrical and asymmetrical stretching vibrations, respectively. The occurrence of the band at 1346 cm^−1^ comes probably from bending vibrations of C–H or O–H groups, while the presence of distinct bands observed together in the range of 1240–900 cm^−1^ is associated with C–O–C stretching vibrations or with –CH–OH in aliphatic cyclic secondary alcohol. The signal at 1067 cm^−1^ implies from overlapping of the bands coming from C–O stretching and OH bending vibrations present in CH–OH groups [25,28,38,39,40,41,42,43]. 

Addition of crosslinker (MBA) and AA-SA copolymer to pectin led to significant changes in IR spectra (lines P1-P6) as a result of the reaction between pectin and AA-AS copolymer in the presence of crosslinker. Apparent weakening of the bands located in the range of ca 3300–3600 cm^−1^ allows us to suppose that hydroxyl groups coming from pectin react with AA-SA copolymer and/or crosslinker, thus their amount decreases. The maximum at 3455 cm^−1^ probably comes from N-H stretching vibrations. The band at 1720 cm^−1^ may be attributed to an asymmetric C=O stretching vibrations present either in COOCH_3_ or carboxylic ions (COO^-^). The bands at ca 1558 and 1453 cm^−1^ may be assigned to amide II and scissoring -CH_2_- vibrations, respectively. 

Another effect of the reaction of crosslinker and AA-SA copolymer with pectin was the appearance of bands at 1409 and 1170 cm^−1^, which reflects -CH_2_- bending and stretching vibrations of C=C in (C=C)–(C=O), respectively. The maximum at 830 cm^−1^ might correspond with bending vibrations coming from carboxylic ions. The presence of the band at 763 cm^−1^ may be associated with rocking vibrations of –CH_2_– groups. Increasing amount of crosslinker in relation to pectin and AA-SA copolymer did not change appearance of the IR spectra (line P1-P6).

In Figure 7b we present the IR spectra of the mixture of pectin, crosslinker and AA-SA copolymer with different chemical composition (line P3A-P3G). The content of crosslinker was constant. In the case of hydrogel P3A, where pectin was not present in the reaction mixture, (Figure 7b, line P3A), the band at 3435 cm^−1^ is typical of –NH– stretching vibrations, meanwhile the signal at 2954 cm^−1^ might be assigned to stretching vibrations of C-H groups (not shown). The distinct band at 1720 cm^−1^ originates from C=O stretching vibration, typical of amide group. The maxima found at 1558 and 1453 cm^−1^ comes from amide II and scissoring –CH_2_– vibrations, respectively. The presence of bands at 1409 and 1170 cm^−1^ is typical of –CH_2_– bending and stretching vibrations of C=C in (C=C)–(C=O), respectively.

At first sight, the IR spectra obtained for the mixture based on MBA, AA-As copolymer and pectin (Figure 7b, line P3B-P3G) seemed to be similar to the IR spectrum of the system not containing pectin (Figure 7b, line P3A). Profound analysis exhibited relevant differences between spectra in the range of 1240–763 cm^−1^. 

Due to increasing content of pectin in the mixture (Figure 7b, line P3B-P3G), it was observed significant growing intensity of the band at 1020 and 1240 cm^−1^ connected with decrease of the maximum at 1170 cm^−1^. It may be concluded that (C=C)–(C=O) groups present in crosslinker undergo reaction with OH groups coming from pectin, and C–O–C groupings are formed. Elevated content of pectin in discussed systems led to the separation of the band at ca 807 cm^-1^ (coming from out-of-plane bending C=C–H vibration in crosslinker) between signals at 830 and 763 cm^−1^ (present mainly in AA-SA copolymer). The presence of the former band is connected with bending vibrations of carboxylic ions, while the latter band may be assigned to rocking vibrations of –CH_2_– groups [25,28,38,39,40,41,42,43].

## 4. Conclusions

In this work, pectin-based hydrogels with excellent swelling properties have been prepared by free radical polymerization of partially neutralized acrylic acid and high methylated (D_e_=67%) pectin. In parallel with the polymerization reaction, cross-linking occurred due to the presence of a cross-linking agent. The structural changes in pectin-based hydrogels have been investigated by FTIR spectroscopy. It was clearly evident that poly(acrylic acid) chains were grafted of pectin particles. GPC analysis of soluble fractions showed that pectin is not present in those fractions, therefore it should be noted that all of the pectin has been incorporated into the hydrogel structure. From these studies, it could be concluded that swelling capacity of hydrogels was dependent on MBA and pectin content. Increasing the content of the crosslinking agent causes a reduction in swelling. However introduction of small amount of pectin (up to 6.7 wt %) to poly(acrylic acid) hydrogel increase swelling capacity. Further increasing of pectin ratio cause decrease of swelling. This study could not only give useful information about structure and swelling parameters of poly(acrylic acid)—high methylated pectin hydrogels, but it is the foundation for further research on hydrogels based on acrylic polymers and other polysaccharides.

## Figures and Tables

**Table 1:** Reaction parameters of synthesized poly(acrylic acid)/pectin hydrogels.

Sample	Pectin, g	MBA, g	AA, g
P1	5	0.0048	30
P2		0.0098	
P3		0.0188	
P4		0.0293	
P5		0.0504	
P6		0.0804	
P3A	0	0.0188	
P3B	1		
P3C	2		
P3D	3		
P3E	5		
P3F	7		
P3G	10		

**Table 2:** Molecular masses of sol fractions of poly(acrylic acid) hydrogels based on high methylated pectin.

Sample	*M*_n_ × 10^−5^, g/mol	*M*_w_ × 10^−5^, g/mol	Ɖ
Pectin	0.88	7.60	8.6
P1	0.55	1.39	2.5
P2	0.40	0.92	2.3
P3	0.54	1.55	2.8
P4	0.48	1.60	3.3
P5	0.85	2.92	3.4
P6	0.73	2.97	4.1
P3A	0.53	1.66	3.1
P3B	0.58	1.94	3.4
P3C	0.61	2.05	3.4
P3D	0.84	3.45	4.1
P3E	0.54	1.63	3.0
P3F	0.73	3.33	4.6
P3G	0.71	2.63	3.7

**Table 3:** Parameters characterized the glass transition process.

Sample	Pectin, g/100 g	MBA, g/100 g	*T*_ON,_ °C	*T*_MID,_ °C	*T*_INF,_ °C	*T*_END,_ °C	*T*_END_—*T*_ON,_ °C	Δ*C*_P,_ J·g^−1^·K^−1^
P1	5	0.0048	66.7 ± 0.2 ^c^	74.9 ± 0.1 ^d^	75.6 ± 0.1 ^c^	81.5 ± 0.2 ^c^	14.8 ± 0.3 ^bc^	0.613 ± 0.009 ^c^
P2	5	0.0098	69.9 ± 0.2 ^e^	79.0 ± 0.1 ^j^	80.0 ± 0.3 ^g^	86.5 ± 0.2 ^g^	16.6 ± 0.3 ^e^	0.660 ± 0.001 ^d^
P3	5	0.0188	67.0 ± 0.1 ^c^	75.4 ± 0.0 ^e^	76.3 ± 0.1 ^d^	82.5 ± 0.1 ^d^	15.5 ± 0.2 ^cd^	0.615 ± 0.000 ^c^
P4	5	0.0293	64.8 ± 0.0 ^b^	73.0 ± 0.0 ^b^	73.6 ± 0.2 ^a^	79.7 ± 0.1 ^b^	14.9 ± 0.1 ^bc^	0.621 ± 0.011 ^c^
P5	5	0.0504	73.7 ± 0.7 ^f^	82.9 ± 0.1 ^k^	84.1 ± 0.1 ^h^	90.4 ± 0.2 ^h^	16.7 ± 0.7 ^e^	0.672 ± 0.004 ^d^
P6	5	0.0804	63.4 ± 0.3 ^a^	71.9 ± 0.1 ^a^	73.1 ± 0.3 ^a^	78.9 ± 0.0 ^a^	15.5 ± 0.3 ^cd^	0.621 ± 0.001 ^c^
P3A	0	0.0183	78.1 ± 0.1 ^g^	85.0 ± 0.0 ^l^	86.7 ± 0.1 ^i^	91.1 ± 0.1 ^i^	13.1 ± 0.2 ^a^	0.742 ± 0.009 ^g^
P3B	1	0.0181	69.3 ± 0.1 ^e^	77.1 ± 0.1 ^h^	78.4 ± 0.1 ^f^	83.4 ± 0.1 ^ef^	14.1 ± 0.2 ^b^	0.715 ± 0.010 ^f^
P3C	2	0.0185	69.7 ± 0.2 ^e^	77.4 ± 0.0 ^i^	78.0 ± 0.2 ^f^	83.8 ± 0.1 ^f^	14.1 ± 0.2 ^b^	0.690 ± 0.004 ^e^
P3D	3	0.0185	68.0 ± 0.2 ^d^	75.7 ± 0.1 ^f^	76.9 ± 0.4 ^e^	82.2 ± 0.4 ^d^	14.2 ± 0.4 ^b^	0.665 ± 0.002 ^d^
P3E	5	0.0188	67.0 ± 0.1 ^c^	75.4 ± 0.0 ^e^	76.3 ± 0.1 ^d^	82.5 ± 0.01 ^d^	15.5 ± 0.2 ^cd^	0.615 ± 0.0000 ^c^
P3F	7	0.0181	67.1 ± 0.1 ^c^	76.1 ± 0.1 ^g^	76.9 ± 0.0 ^e^	83.4 ± 0.1 ^e^	16.3 ± 0.2 ^de^	0.577 ± 0.010 ^b^
P3G	10	0.0178	65.0 ± 0.2 ^b^	74.4 ± 0.2 ^c^	75.1 ± 0.2 ^b^	81.7 ± 0.1 ^c^	16.8 ± 0.3 ^e^	0.504 ± 0.010 ^a^
One-way ANOVA—*p*			<0.001	<0.001	<0.001	<0.001	<0.001	<0.001

Differences between values signed the same letters in particular columns are non-significant at 0.05 level of confidence.

**Table 4:** Parameters characterized the melting process.

Sample	Pectin [g/100 g]	MBA [g/100 g]	*T*_ON,_ °C	*T*_P,_ °C	*T*_END,_ °C	*T*_END_—*T*_ON,_ °C	−Δ*H*, J·g^−1^
P1	5	0.0048	189.5 ± 1.0 ^abcd^	192.2 ± 1.1 ^bc^	200.0 ± 0.6 ^b^	10.5 ± 0.5	185.6 ± 10.0 ^cd^
P2	5	0.0098	191.1 ± 1.3 ^bcde^	193.7 ± 1.1 ^bcde^	200.8 ± 1.7 ^b^	9.7 ± 0.6	173.2 ± 1.3 ^e^
P3	5	0.0188	188.4 ± 0.6 ^ab^	191.8 ± 1.0 ^ab^	200.9 ± 0.1 ^bc^	12.5 ± 0.9	183.9 ± 1.6 ^d^
P4	5	0.0293	188.1 ± 1.0 ^ab^	191.3 ± 1.1 ^ab^	200.9 ± 0.2 ^b^	12.8 ± 1.3	194.1 ± 2.3 ^b^
P5	5	0.0504	192.6 ± 0.9 ^de^	195.1 ± 1.1 ^de^	202.2 ± 0.5 ^bc^	9.6 ± 1.6	163.8 ± 2.7 ^f^
P6	5	0.0804	189.3 ± 1.8 ^abc^	192.3 ± 2.1 ^bcd^	200.5 ± 1.7 ^b^	11.3 ± 0.4	193.9 ± 2.3 ^bc^
P3A	0	0.0183	197.8 ± 0.5 ^f^	200.4 ± 0.6 ^f^	207.9 ± 0.3 ^d^	10.1 ± 0.3	120.2 ± 0.1 ^h^
P3B	1	0.0181	193.8 ± 1.6 ^e^	196.2 ± 1.2 ^e^	203.7 ± 1.6 ^c^	10.0 ± 0.4	143.9 ± 6.0 ^g^
P3C	2	0.0185	192.3 ± 0.1 ^cde^	194.8 ± 0.3 ^cde^	201.9 ± 1.7 ^bc^	9.6 ± 2.0	159.0 ± 3.5 ^f^
P3D	3	0.0185	189.1 ± 2.6 ^ab^	191.9 ± 3.3 ^b^	199.8 ± 0.9 ^b^	10.8 ± 2.5	165.7 ± 1.8 ^ef^
P3E	5	0.0188	188.4 ± 0.6 ^ab^	191.8 ± 1.0 ^ab^	200.9 ± 0.1 ^b^	12.5 ± 0.9	183.9 ± 1.6 ^d^
P3F	7	0.0181	189.8 ± 0.6 ^bcd^	192.4 ± 0.1 ^bcd^	200.2 ± 1.9 ^b^	10.4 ± 2.0	184.0 ± 0.3 ^d^
P3G	10	0.0178	186.4 ± 1.7 ^a^	188.9 ± 1.8 ^a^	196.55 ± 1.9 ^a^	10.15 ± 0.0	203.8 ± 2.2 ^a^
One-way ANOVA—*p*			<0.001	<0.001	<0.001	0.307	<0.001

Differences between values signed the same letters in particular columns are non-significant at 0.05 level of confidence.
